# Effect of United States buckwheat honey on antibiotic-resistant hospital acquired pathogens

**DOI:** 10.11604/pamj.2016.25.212.10414

**Published:** 2016-12-06

**Authors:** Eric Nee-Armah Hammond, Megan Duster, Jackson Ssentalo Musuuza, Nasia Safdar

**Affiliations:** 1 Institute for Clinical and Translational Research, University of Wisconsin-Madison, Madison, WI 53705, USA; 2 Division of Infectious Diseases, Department of Medicine, University of Wisconsin School of Medicine and Public Health, Madison, WI 53705, USA; 3 William S. Middleton Memorial VA Medical Center, Madison WI 53705, USA

**Keywords:** Buckwheat honey, susceptibility, antibiotic-resistant, nosocomial pathogen

## Abstract

**Introduction:**

Due to an upsurge in antibiotic-resistant infections and lack of therapeutic options, new approaches are needed for treatment. Honey may be one such potential therapeutic option. We investigated the susceptibility of hospital acquired pathogens to four honeys from Wisconsin, United States, and then determined if the antibacterial effect of each honey against these pathogens is primarily due to the high sugar content.

**Methods:**

Thirteen pathogens including: four *Clostridium difficile*, two Methicillin-resistant *Staphylococcus aureus*, two *Pseudomonas aeruginosa*, one Methicillin-Susceptible *Staphylococcus aureus*, two Vancomycin-resistance Enterococcus, one *Enterococcus faecalis* and one *Klebsiella pneumoniae* were exposed to 1-50% (w/v) four Wisconsin honeys and Artificial honey to determine their minimum inhibitory concentration (MIC) and minimum bactericidal concentration (MBC) using the broth dilution method.

**Results:**

Buckwheat honey predominantly exhibited a bactericidal mode of action against the tested pathogens, and this varied with each pathogen. *C. difficile* isolates were more sensitive to the Wisconsin buckwheat honey as compared to the other pathogens. Artificial honey at 50% (w/v) failed to kill any of the pathogens. The high sugar content of Wisconsin buckwheat honey is not the only factor responsible for its bactericidal activity.

**Conclusion:**

Wisconsin buckwheat honey has the potential to be an important addition to therapeutic armamentarium against resistant pathogens and should be investigated further.

## Introduction

Antibiotic-resistant pathogens (Methicillin-resistant *Staphylococcus aureus* (MRSA), *Pseudomonas aeruginosa, Klebsiella pneumoniae, Clostridium difficile* and *Enterococcus faecalis*) are major causes of severe infections in hospitalized patients leading to longer hospital stays and higher mortality rates worldwide [1, 2]. In the United States, infections associated with antibiotic-resistant organisms occur in over 2 million people and at least 23,000 deaths are recorded annually [3]. According to the WHO report [2], people infected with MRSA are reported to be 64% more likely to die than those infected with antibiotic sensitive strains.

Treatment of antibiotic-resistant associated infection is challenging particularly in healthcare settings due to the increasing trend of antibiotic resistance, side effects of important antibiotics, limited antibiotic options [1, 2] and reduction in new antibiotic discovery endeavors by pharmaceutical companies [2, 4]. Alternative therapeutic interventions that are effective and without adverse effects are urgently needed. Honey is one such promising option.

Natural honey is obtained from nectar collected by honeybees. Its high sugar content coupled with low pH, bee-derived enzymes, bee-derived peptides and phytochemical compounds contribute to its antibacterial action [5–11]. Honey also has antioxidant, anti-inflammatory and anti-hyaluronidase properties which vary depending on the nectar source [5, 12, 13]. The amount of natural phenol in honey plays a significant role in its inhibition activity [12, 13]. Honeys with high concentration of phenol are more likely to possess high inhibitory efficacy than those with low or no phenol.

The use of honey as medicinal remedy was initiated many centuries ago, but recent publications have demonstrated the antibacterial efficacy of honey in *in vitro* and *in vivo* [14–22]. The antibacterial mechanism of honey has gradually been unraveled [23]. Evidence from published studies show that honey disrupts cell walls in *P. aeruginosa* [23] and interrupts cell division in MRSA [24]. Honey also stimulates inflammatory cytokines [25] and has been identified to be a strong scavenger of super peroxide anions and highly effective inhibitor of reactive oxygen species (ROS) stimulated from human polymorphonuclear neutrophils (PMNs) [5]. Both medical grade honey and raw honey have been shown to have broad spectrum antibacterial activity against a plethora of pathogens, including antibiotic-resistant organisms and their biofilms [19, 26, 27]. Furthermore, honey has been shown to heal recalcitrant wounds [28–31]. Effective application of honey promotes wound healing, prevents cross-infection, and repairs tissue [32]. Unlike some antimicrobial agents (such as fluoroquinolones and clindamycin), honey has no record of adverse side effect on tissues [32] or gut [21].

Public interest in the therapeutic use of natural honey in recent times has greatly increased [31]. Licensed medical grade honeys are available in the medical field globally but the majority are derived from *Leptospermum species* found in Australia and New Zealand. The antibacterial property of medical grade Manuka honey is highly recognized in the research field due to its high unique property, Unique Manuka factor (UMF). Though much work has been done on honey, little is known about the antibacterial efficacy of honey from the United States. In this pilot study, our goals were to compare the efficacies of American honeys and artificial honey on antibiotic-resistant pathogens and then determined if the antibacterial effect of each honey against these pathogensis primarily due to its high sugar content.

## Methods

### Types of honey

Four Wisconsin honeys (Buckwheat honey, Wild honey, Cranberry honey and Orange blossom honey) and an artificial honey (AH) were studied. Wisconsin honeys were locally produced in Wisconsin, USA, and purchased from a local grocery supermarket. AH (sugar solution) 100 g was comprised of 7.5 g D-(+)-maltose monohydrate, 40.5 g D-(-)-fructose, 1.5 g sucrose, and 33.5 g D-(+)-glucose (Sigma-ALDRICH Co., Missouri, USA) dissolved in 17 ml sterile osmotic reverse water [33]. All honeys were stored at 4°C in a dark environment until use.

Stock solution of honey preparation: A stock honey solution of 50% (w/v), was prepared by dissolving 25 g honey in 50 ml Muller-Hinton broth (MHB) (Thermo Fisher Scientific Remel product, Lenexa, KS, USA) in a 50 ml volumetric flask. The stock solution was diluted for further analysis. Concentrations of honeys were expressed as weight/volume percent (% (w/v)).

#### Bacteria strains

A total of 13 pathogens (nine aerobic and four anaerobic) were used in the study. The isolates were selected based on their toxigenicity and clinical significance. The pathogens used for the study were received from the archived culture collection of the University of Wisconsin-Madison Infectious Disease Research Laboratory. Their sensitivity to different antibiotics is shown in (Table 1).

**Table 1:** Antibiogram characteristics of pathogens

Pathogens	Antibiotics sensitivity	
	Sensitive to	Resistant to
MSSA ATCC 29213	Ciprofloxacin, Cefoxitin, Vancomycin	
MRSA ATCC 33592		Cefoxitin
MRSA 0814^+^		Cefoxitin
*E. feacalis* ATCC 51299	Ciprofloxacin	Vancomycin
VRE 002^+^		Vancomycin
VRE ATCC 51559^+^		Ciprofloxacin, Vancomycin
*P. aeruginosa* 007^+^		Ciprofloxacin
*P. aeruginosa* ATCC 27853	Ciprofloxacin	Cefoxitin
*K. pneumoniae* 90 (ESBL)^+^	Ciprofloxacin	
*C. difficile* (Ribotype 027)	NT	NT
*C. difficile* (Ribotype 087)	NT	NT
*C. difficile* 0001^+^	NT	NT
*C. difficile* 0009^+^	NT	NT

+ Clinical isolates. ESBL=Extended spectrum beta-lactamase–producing organism. NT= not tested.

The aerobic pathogens consisted of two MRSA (ATTC 33592 and 0814) strains, two *P. aeruginosa* (ATCC 27853 and ATCC 51559) strains, one Methicillin-Susceptible *S. aureus* (MSSA) (ATCC 29213) strain, two vancomycin-resistant *Enterococcus* (VRE) (ATCC 51559 and VRE 002) strains, *E. faecalis* ATCC 51299, and *K. pneumoniae*. To ensure viability and purity, each pathogen recovered from a -80°C freezer was sub-cultured on blood agar (BA) plates (Thermo Fisher Scientific Remel product, Lenexa, KS, USA) and incubated aerobically at 37°C for 24 h before susceptibility tests were performed. Overnight cultures were emulsified in phosphate buffered saline (PBS) (Fisher BioReagent, New Jersey, USA) to 0.5 McFarland standard and further diluted in Muller-Hinton Broth-2 (MHB2) (Sigma-ALDRICH Co., Missouri, USA) to approximately 5x10^6^ CFU/ml for immediate testing.

The anaerobic pathogens, four toxigenic *C. difficile* isolates (ATCC BAA-1870 (Ribotype 027), ATCC 43255 (Ribotype 087), 0001 and 0009), were grown in BBL Brucella Broth (SBB) (Becton Dickinson, Sparks, MD) supplemented with hemin (Sigma-Aldrich CO., St. Louis, MO) and vitamin K (Alfa Aesar, Ward Hill, MA) as recommended by the Clinical and Laboratory Standards Institute (CLSI) document M11-A7 [34]. Incubation was carried out in an anaerobic chamber (Coy Laboratory Products, Inc., Grass Lake, MI) with anaerobic mixed gases (10% H_2_, 10% CO_2_, 80% N_2_). Agar plates and reagents were pre-reduced in an anaerobic cabinet overnight before use. Tested organisms were retrieved from a -80°C storage freezer then sub-cultured at 24 h intervals at least three times on BA plates (Remel, Lenexa, KS, USA) and incubated anaerobically to confirm the purity and viability of the organisms before use. Overnight broth cultures were used for each test.

The overnight cultures, which grew heavily, were diluted in PBS (Fisher BioReagent, New Jersey, USA) to 0.5 McFarland standard and further diluted in SBB to approximately 5x10^6^ CFU/ml before further susceptibility tests were performed. Colony count was also monitored on BA plates to ensure all the wells received equal and accurate amount of inoculum density.

### Minimum inhibitory concentration determination

The minimum inhibitory concentration (MIC) was determined in 96 well round-bottomed microtiter plates (Corning Incorporated, New York, USA). Broth microdilution method was used according to CLSI guidelines [34] and [35] with modifications. A volume of 100 µl of varied honey concentrations (0-50%) (w/v) was distributed in wells 1-10. Wells 11 and 12 were considered positive (broth) and negative controls (broth and honey only), respectively. Subsequently, wells 1-11 were seeded with an aliquot of 10 µl of approximately 10^6^ CFU/mL of overnight MHB culture and incubated at 37°C for 24 h aerobically. Positive control and negative control were added to monitor viability and sterility of honey, respectively. Reference stains were also included to monitor consistency. Wells with the lowest honey concentrations which prevented growth/turbidity under a magnifying mirror were considered as MIC. For quality assurance purposes, each experiment was run in triplicate at different occasions. The method was validated by using standard antibiotics against the reference strains and the results compared to that in CLSI literature.

### Minimum bactericidal concentration determination

Minimum bactericidal concentration (MBC) was determined by plating 10 µl of content from all the MIC wells without visible growth/turbidity onto antibiotic-free or honey-free BA plates in duplicate and incubating aerobically and anaerobically as required. Positive and negative control wells were included. The lowest honey concentration that killed the organism was considered the MBC. MBCs were determined on three separate occasions.

### Statistical analysis

Data was entered into Microsoft Excel^®^ 2010 and analyzed. The Student´s t-test was performed to determine whether the differences in mean of Wisconsin Buckwheat honey (WBH) and the AH for each isolate were significant. A p-value ≤ 0.05 was considered significant. For each honey type, the ratio of MBC to MIC was determined and used to classify the antibacterial activity of the honey as either bactericidal or bacteriostatic. Bactericidal was defined as MBC/MIC ratio less than or equal to 4, while a MBC/MIC ratio above 4 and less than 16 was considered bacteriostatic [36].

## Results

**Figure 1:**
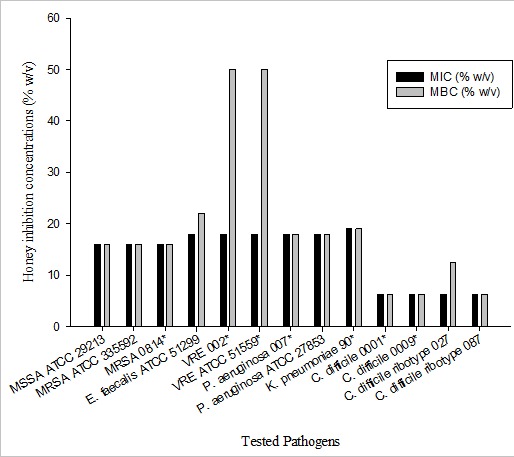
The minimum inhibitory concentrations (MIC) and minimum bactericidal concentrations (MBC) (% w/v) of WBH, on antibiotic-resistant pathogens. The values are represented as mean of triplicate results. The investigation of MIC and MBC values for aerobic and anaerobic isolates were carried out after 24 h and 48 h incubation respectively. American Type Culture Collection (ATCC). +Clinical isolates. ++ MBC >50.

The selected pathogens for the study are listed in Table 1. The MICs and MBCs of the selected pathogens are shown on (Figure 1). Generally, WBH exhibited bactericidal mode of action against all the tested organisms with MBC/MIC ≤ 4. WBH exhibited a broad spectrum antimicrobial property but Gram-positive organisms were slightly more susceptible than Gram-negative organisms (Figure 1). There was a dose response relationship between the tested honey and the pathogens. The MICs of the natural honey was very low as compared to that of the AH. For the aerobic organisms, AH inhibited growth at 50% (w/v) whiles that for anaerobic organisms was 40% (w/v). These high concentrations failed to kill any of the organisms. However, WBH demonstrated bactericidal activity with MIC range between 6.25-19% (w/v) and MBC range between (6.25-50% (w/v) (Figure 1). The effect of WBH on VRE 002 and VRE 51559 were high with MBCs of 50 and >50 (% w/v) respectively. There was a significant difference in susceptibility between the WBH and AH for all the tested isolates (p<0.05). The cranberry honey, wild honey and orange blossom honey did not exhibit bactericidal activity at 50% (w/v).

## Discussion

The rise in multidrug and extreme antibiotic-resistant pathogens in the healthcare settings is so alarming that it has become necessary to find alternative and effective natural therapeutic agents. In this study, we tested the potency of four local honeys against standardized pathogens (MRSA, *P. aeruginosa*, MSSA, VRE, *E. faecalis*, *K. pneumoniae* and *C. difficile*). Honeys that did not exhibit bactericidal activity at 50% (w/v) during the study were excluded.

Our study demonstrated that MICs of WBH for the four *C. difficile* isolates were the same (6.25 % w/v) with MBC ranging between 6.25-12.5% (w/v). Despite the different nectar sources of the honeys, these results were congruent with previous work by Hammond and Donkor [17] who demonstrated that Woundcare Manuka honey inhibited *C. difficile*. The striking antibacterial activity of WBH on *C. difficile* is an evidence that some natural honeys have the potential to treat *C. difficile* infection. These honeys could be developed for oral administration and could represent a big step forward in the treatment of antibiotic-resistant nosocomial infections.

Additionally, WBH exhibited bactericidal effects on MRSA, VRE, *E. faecalis, K. pneumoniae*, and *P. aeruginosa* with MIC range 16-20 (% w/v) and MBC range 16-50 (% w/v). A similar study by Brudzynski et al. [37] indicated that Canadian buckwheat honey displayed a powerful bactericidal effect against antibiotic-resistant pathogens. Cooper et al [38] also established that the effect of Medihoney on *P. aeruginosa* ATCC 27853 was bactericidal with MIC of 15.7% (w/v). In comparison, some of our results varied from those obtained by other investigators. Factors responsible for these discrepancies may include change in honey activity level from batch to batch, and materials, methods, and techniques employed. These findings add up to previous results obtained on susceptibility of multiple organisms to honey of different nectar sources and geographical origin [15, 16, 19, 22, 33, 37, 39].

To investigate the role of high sugar content of selected honeys on antibacterial activity, we exposed test pathogens to various concentrations of AH (10-50% w/v) to mimic the main sugar composition in natural honeys. Our findings indicated that a high AH concentration (50% w/v) failed to completely prevent any of the pathogens from growth, whereas WBH exhibited bactericidal activity at very low honey concentrations (6.25% w/v). A similar conclusion was reported by [16]. Likewise at 50% (w/v), cranberry honey, wild honey and orange blossom honey supported the growth of all the studied pathogens. This further confirms that bactericidal activity of honey is not solely due to the presence of high sugar content and that varying potent antibacterial compounds in honey may work synergistically to extensively disrupt cells and lysis of pathogens as reported by Henriques et al. [23]. This study suggests that WBH may have a complex composition that could effectively resist multiple antibiotic-resistant pathogens to thrive.

WBH remarkably displayed broad spectrum antimicrobial activity in this study. Other published research have demonstrated that phenolic compound in buckwheat honey is high and this influences its antibacterial property [5, 12, 13]. Based on this, we can deduce that the phenol content in Wisconsin honey may be high, and hence could be a major factor contributing to its antibacterial activity against these important hospital acquired pathogens. The potent bactericidal effect of WBH suggests that more honeys need to be tested to supplement existing medical grade honeys and standard antibiotics to effectively control the increasing antibiotic-resistant pathogens during therapy. If processed, WBH could be employed therapeutically. Furthermore, buckwheat honey is common in the United States and may be cost effective than imported medical grade Manuka honey but further study is required.

Our findings suggest that it is important to carry out further investigations to determine the rate and concentrations at which WBH inhibits pathogens. Investigating the effect of WBH on *C. difficile* spores could be advantageous since *C. difficile* infections mode of transmission is also through spores which can survive for a long time in the contaminated environment. Since microbial biofilm delays wound healing, it is important to determine if WBH prevents or disrupts biofilm formed by pathogens in the study that could possibly cause wound infections. Furthermore, it will be essential to determine if the antibacterial efficacy of the selected buckwheat honey is representative of all buckwheat honeys in the whole state of Wisconsin or the United States.

## Conclusion

We provide the first data on antibacterial efficacy of buckwheat honey from Wisconsin, USA against nosocomial or hospital acquired pathogens. Our data demonstrate that antibiotic and multiple drug resistant pathogen(s) were susceptible to WBH. We also deduced that the antibacterial effect of WBH on pathogens including *C. difficile* was not mainly due to its high sugar content. Future work should include *in vivo* studies to examine efficacy and mechanism of action.

### What is known about this topic

- Antibiotic resistant pathogens is a major cause of deaths in hospitals;
- Antibacterial activity of honey from different nectar sources differ;
- High sugar content in honey is not solely responsible for antibacterial activity of honey.

### What this study adds

- Wisconsin buckwheat honey has bactericidal activity against antibiotic-resistant pathogens;
- This is the first data on Wisconsin buckwheat honey against antibiotic-resistant pathogens including *Clostridium difficile*;
- Wisconsin buckwheat honey has the potential to treat nosocomial associated infections.
